# A 32-Year-Old Female with AIDS, *Pneumocystis jiroveci* Pneumonia, and Methemoglobinemia

**DOI:** 10.1155/2013/980589

**Published:** 2013-06-27

**Authors:** Guillermo J. Giangreco, Dean Campbell, Mark J. Cowan

**Affiliations:** ^1^Baltimore-Washington Medical Center, Glen Burnie, MD 21061, USA; ^2^University of Maryland School of Medicine, Baltimore, MD 21201, USA

## Abstract

We report a case of methemoglobinemia with significant hemoglobin desaturation in a young female with AIDS who was being treated for *Pneumocystis jiroveci* pneumonia. A review of the etiology, pathophysiology, and treatment of methemoglobinemia is presented.

## 1. Background

Methemoglobinemia is the presence of a significant amount of oxidized iron (Fe^3+^, met-Hgb) within hemoglobin (Hgb) in the blood, rendering it unable to bind oxygen. It is caused by a number of medications and toxins and can quickly degrade oxygen transport sufficiently enough to cause or aggravate severe tissue hypoxemia. Its hallmark features are hemoglobin desaturation out of proportion to blood partial pressure of oxygen and “chocolate brown” blood. Definitive diagnosis can be made quickly and easily with co-oximetry, but must be suspected, as co-oximetry is not routinely performed in patients. It is important to recognize, as correction of the pathologic hemoglobin redox state with methylene blue is simple, rapid, effective, and lifesaving. We present a case of methemoglobinemia secondary to primaquine, which was successfully treated with methylene blue and discontinuation of the drug.

## 2. Case Presentation

A 32-year-old female with a history of intravenous drug abuse and AIDS (last CD_4_ count = 26/mm^3^) was admitted to a local hospital for cough, fever, and respiratory distress. Initial blood cultures grew gram-positive cocci in clusters, and endocarditis was suspected. Vancomycin was started, but a transthoracic echocardiogram was nondiagnostic, and the patient was transferred to our institution for further evaluation.

 The patient was unmarried and had been HIV positive for three years. She had no history of opportunistic infections and had been in good health for the past year taking no medications. She smoked one pack of cigarettes/day, used heroin intravenously 3–5 times/week, and denied alcohol use. She reported an intolerance to sulfa drugs.

On arrival, we found a slender female in moderate to severe respiratory distress. Temperature was 99.2°F, respiratory rate of 35/min, blood pressure 110/62 mmHg, and heart rate 140/minute. Oxygen saturation measured by pulse oximetry was 88% on a 100% nonrebreather mask. She could speak only in short sentences. Cardiac examination revealed tachycardia without murmur. Lung examination revealed coarse crackles throughout. Abdomen was benign and the extremities were without cyanosis or edema. Skin showed evidence of chronic intravenous needlesticks without cellulitis. The remainder of the physical examination was unremarkable.

Arterial blood gases obtained on presentation showed a pH of 7.51, pCO_2_ of 33 mmHg, and a pO_2_ of 59 mmHg with a saturation of 89% on room air. White blood cell count was 4,300 cells/*μ*L, hemoglobin was 10.4 g/dL, and the hematocrit was 35%. Electrolytes were within normal limits; serum LDH was 654 IU/L. Chest X-ray showed diffuse bilateral airspace disease with an upper lobe predominance. G6PD level was normal.

She was started on empiric gatifloxacin, primaquin, clindamycin, and prednisone for community-acquired pneumonia, with coverage for *Pneumocystis jiroveci*. The patient respiratory status worsened despite noninvasive ventilation, and she required intubation and mechanical ventilation on hospital day 5. A diagnostic bronchoscopy with broncho-alveolar lavage was performed, demonstrating *Pneumocystis jiroveci* cysts. Gatifloxacin was discontinued, and the patient experienced improvement of her hypoxemia over the next day (FIO_2_ of 60% and PEEP of +8). On day 7, she developed moderate digital and perioral cyanosis and an increasing lactic acid level (7.3 mmol/L). ABG showed pH 7.42, pCO_2_ 33, with a saturation of only 86% despite a pO_2_ of 165. Co-oximetry revealed a carboxyhemoglobin level of 1.6% and a methemoglobin level of 20.1%. She received methylene blue 100 mg IV and was switched from primaquin to intravenous pentamidine. Methemoglobin did not recur. She ultimately died from complications of multiorgan system failure (MOSF) on hospital day 12.

## 3. Discussion

**Figure 1:**
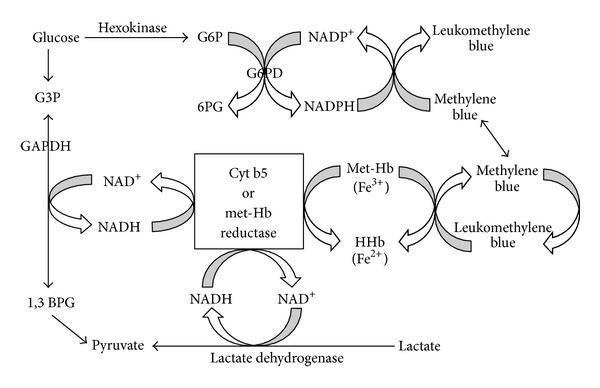
Biochemical pathways of hemoglobin reduction. NAD Nicotine adenine dinucleotide, GAPDH: glyceraldehyde-3-phosphate dehydrogenase, G3P: glyceraldehyde-3-phosphate, 1,3 BPG: 1,3 bisphosphoglycerate, cyt: cytochrome, and metHB: methemoglobin. Adapted from [6].

Met-Hgb is Fe^3+^ in the heme moiety of Hgb. Normally, met-Hgb is produced at low levels by oxidative stress in the blood at a rate of 3% per day [1]. Fe^+3^ is rapidly and efficiently reduced to normal (Fe^+2^) via the cytochrome b5 reductase pathway (see Figure 1 for the heme redox cycle), normally keeping met-Hgb levels <1% [2]. Backup reduction is provided by nicotinamide adenine dinucleotide phosphate (NADPH)-met-Hgb reductase, which in turn requires the glucose-6-phosphatase (G6P)/glutathione reductase system to maintain NADPH levels. This pathway typically accounts for only 5% of met-Hgb reduction, as it is primarily used in reducing oxidant xenobiotics rather than met-Hgb [3]. Met-Hgb will not normally increase to clinically significant levels unless production is increased (acquired disease), or reduction is decreased (hereditary disease) [4, 5].

The inherited types of methemoglobinemia are associated with enzymatic deficiencies in pathways that reduce met-Hgb to Hgb, or with an abnormal Hgb (M type) that resists reduction. Due to the chronic nature of the inherited type, compensatory mechanisms such as increased red blood cell mass and cardiac output have time to develop, and thus patients are characteristically cyanotic but asymptomatic [1, 5].  Table 1 lists the causes of inherited met-Hgb.

Acquired met-Hgb results from exposure to drugs, pollutants, and toxins, which are also listed in Table 1. The common causative agents in hospitalized patients include local anesthetics, sulfa antibiotics, dapsone, primaquin, nitrates, and metoclopramide. These generally induce methemoglobinemia by increasing the oxidation rate of Hgb by 100-fold or greater, overwhelming the blood's reductase systems and driving NADPH to very low levels [1, 3, 5]. Dapsone is an antimicrobial and anti-inflammatory agent that has also been shown to cause methemoglobinemia. Met-Hgb levels as high as 55% have been reported in patients after dapsone overdose [15, 16]. Primaquin is more likely to cause met-Hgb when the daily dose exceeds 60 mg [17].

Mild cyanosis is characteristic of methemoglobinemia. Only about 1.5 g/dL (10–15%) of met-Hgb is needed to produce detectable cyanosis, and up to 70% met-Hgb can be relatively well tolerated if the amount of Hgb is adequate, and there has been enough time to develop compensatory mechanisms. Met-Hgb levels of 25–50% can cause headache, confusion, and chest pain [18]. More problematic is acquired met-Hgb in a critically ill patient. Cyanosis can easily be missed or overlooked in ICU patients for a variety of reasons: poor illumination of the room, dark skin, and incorrect attribution of the cyanosis to another cause [19]. In addition to decreasing the amount of normal Hgb available to deliver oxygen to the tissues, the presence of met-Hbg increases the O_2_-Hgb affinity within the affected multimer, left shifting the Hgb dissociation curve. This further decreases oxygen delivery by decreasing oxygen offloading in peripheral tissues [19, 20]. Many critically ill patients are already either hypoxemic or have insufficient oxygen delivery and are frequently exposed to drugs capable of inducing met-Hgb. An otherwise well tolerated level of met-Hgb can manifest symptoms and signs which reflect body-wide inadequate oxygen delivery including myocardial ischemia/infarction, hemodynamic instability, lactic acidosis, ischemic bowel, and stroke. We suspect that drug-acquired met is underappreciated in the ICU setting and may contribute to adverse clinical outcomes.

**Figure 2:**
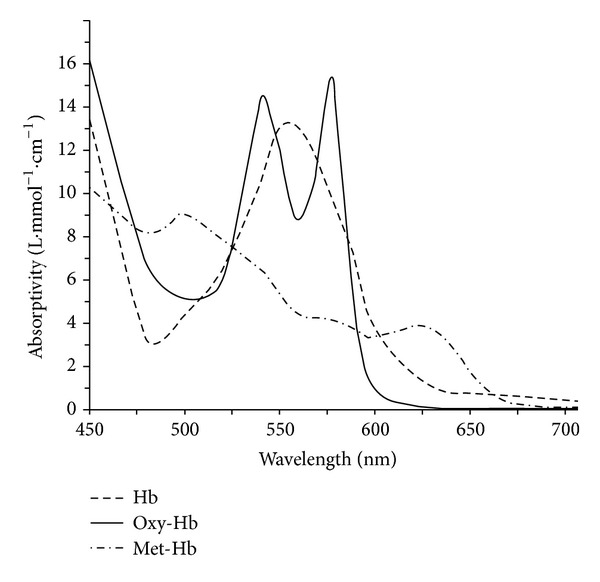
Absorbance as a function of wavelength for oxyhemoglobin, reduced hemoglobin, and methemoglobin. Transmission pulse oximetry utilizes absorbance at two wavelengths (here red 660 nm and infrared 910 nm) to determine the oxygen saturation of an arterial pulsation of blood into the skin. Adapted from [25].

Pulse oximetry, especially in combination with arterial blood gas analysis, is useful in the diagnosis of methemoglobinemia. The absorption spectrum of met-Hgb overlaps with that of oxyhemoglobin at 660 and 910 nm, the wavelengths used in most pulse oximeters (Figure 2), and so direct measurement of met-Hgb with pulse oximetry is impossible [21]. However, there are features that should raise the concern for the presence of met-Hgb. In the presence of met-Hgb, the measured O_2_ saturation will be decreased from that predicted by the PaO_2_ and a normal oxygen-Hgb dissociation curve. A rule of thumb is that the saturation, as measured by pulse-oximetry, will drop by one half of the met-Hgb concentration between 3 and 20%. Thus, a patient with 98% saturated arterial hemoglobin who develops a met-Hgb level of 10% can be expected to have pulse oximetry of 93% [22]. With higher levels of met-Hgb (>30%), the pulse oximetry tends to plateau around 85%, irrespective of true oxygen content or met-Hgb levels, and the arterial blood is characteristically chocolate brown in color [22, 23]. Definitive measurement of met-Hgb requires co-oximetry, a test which is not performed routinely in most ICUs, and must be ordered specifically. Co-oximetry uses multiple wavelengths of light to correctly distinguish met-Hgb from oxyhemoglobin, deoxyhemoglobin, and carboxyhemoglobin [22, 24]. 

Initial treatment consists of discontinuation of the offending drug. Methylene blue, a dye that acts to transport an electron from NADPH to hemoglobin, may be administered at a rate of 1-2 mg/kg for 5 minutes to hasten met-Hgb reduction, although in theory other antioxidants such as vitamin C (risk of renal stones and hyperoxaluria), tocopherol, or N-acetyl cysteine may also be beneficial, and have been utilized in occupational and congenital diseases [18, 19]. Of note, due to a severe hemolytic reaction, methylene blue therapy is contraindicated in G6PD deficient patients. Methylene blue is recommended in symptomatic patients with met-Hgb levels >20%, and in asymptomatic patients with levels >30% [18, 23].

### 3.1. Follow Up

Methylene blue 1.5 mg/kg was administered intravenously, causing the patient's pulse oximetry to increase to 93% after 1-2 minutes and with immediate resolution of her cyanosis. Repeat co-oximetry demonstrated complete resolution of the met-Hgb. She was switched from primaquin-clindamycin to IV pentamidine to complete her therapy for PCP. While her met-Hgb never recurred, she did not survive her hospitalization. Supportive care was withdrawn after the development of severe MOSF. We suspect that methemoglobinemia may have played a role in her progression to MOSF, by converting a borderline hypoxemia into a profound deficiency in tissue oxygen delivery during her critical illness. 

## 4. Conclusion

Many drugs routinely used in the ICU can cause methemoglobinemia, adding to deficient oxygen delivery in this vulnerable patient population. A high index of suspicion should be kept for methemoglobinemia in ICU patients. Clues to the presence of met-Hgb are cyanosis, chocolate brown blood, and dissociation between expected (from paO_2_) and measured Hgb saturation, especially when pulse oximetry reads about 85%. Definitive diagnosis is made by sending an arterial blood sample for co-oximetry. Treatment is removal of the offending drug, with methylene blue administration if tissue hypoxia is critical.

## Figures and Tables

**Table 1:** Causes of methemoglobinemia.

*Congenital *
Abnormal hemoglobin
Hemoglobin M_Boston_
Hemoglobin M_Hyde Park_
Hemoglobin M_Iwate_
Hemoglobin M_Milwaukee_
Hemoglobin M_Ratnagiri_
Hemoglobin M_s_
Hemoglobin M_Saskatoon_
Enzyme deficiency
Cytochrome b5/NADH reductase deficiencies (Types I–IV)
G6PD deficiency
NADPH-flavin reductase deficiency
*Acquired *
Anti-infectives
Chloroquine
Dapsone
Nitrofurans
Primaquine
Rifampin
Sulfanilamide (topical)
Sulfonamides
Sulfoxone
Chemicals
Acetanilide
Alloxan
Aniline derivatives
Aromatic amines
Arsine
Bivalent copper
Chlorates
Chromates
Dimethyl sulfoxide
Dimethyltoluidine
Ferricyanide
Hydroxylamine
Naphthalene
Phenacetin
Toluidine
Phenols
Drugs
Acetaminophen (metabolites)
Clofazimine
Flutamide
Methylene blue (high dose)
Isosulfan blue
Metoclopramide
Nitric oxide
Nitrous oxide
Paraquat
Phenazopyridine
Phenytoin
Rasburicase
Resorcinol
Sodium valproate
Sulfasalazine
Environmental/occupational
Automobile exhaust fumes
Inks
Nitrites
Paints
Propellants
Room deodorizer
Varnishes
Foods
Menthol
Fava beans
Vegetables (spinach, beets, and carrots)
Well water
Local anesthetics
Benzocaine
Bupivacaine
Lidocaine
Prilocaine
Tetracaine (lozenges)
Nitrates
Alkyl nitrate
Amyl nitrate
Bismuth subnitrate
Butyl nitrate
Dinitrophenol
Isobutyl nitrate
Nitrobenzene
Nitroglycerin
Nitrophenol
Nitroprusside
Silver nitrate
Trinitrotoluene

Adapted from [7–14].
